# Status and developmental trends in recombinant collagen preparation technology

**DOI:** 10.1093/rb/rbad106

**Published:** 2023-11-29

**Authors:** Xiaolei Guo, Yuan Ma, Hang Wang, Hongping Yin, Xinli Shi, Yiqin Chen, Guobiao Gao, Lei Sun, Jiadao Wang, Yunbing Wang, Daidi Fan

**Affiliations:** Center for Medical Device Evaluation, National Medical Products Administration, Beijing 100081, China; State Key Laboratory of Tribology, Tsinghua University, Beijing 100084, China; School of Life Science and Technology, China Pharmaceutical University, Nanjing 210009, China; School of Life Science and Technology, China Pharmaceutical University, Nanjing 210009, China; Center for Medical Device Evaluation, National Medical Products Administration, Beijing 100081, China; State Key Laboratory of Tribology, Tsinghua University, Beijing 100084, China; Center for Medical Device Evaluation, National Medical Products Administration, Beijing 100081, China; Center for Medical Device Evaluation, National Medical Products Administration, Beijing 100081, China; State Key Laboratory of Tribology, Tsinghua University, Beijing 100084, China; National Engineering Research Center for Biomaterials & College of Biomedical Engineering, Sichuan University, Chengdu 610064, China; Biotech. & Biomed. Research Institute, Northwest University, Xi'an 710127, China

**Keywords:** recombinant collagen preparation, genetic engineering, collagen characterization, physical transfection, seed cell sorting

## Abstract

Recombinant collagen is a pivotal topic in foundational biological research and epitomizes the application of critical bioengineering technologies. These technological advancements have profound implications across diverse areas such as regenerative medicine, organ replacement, tissue engineering, cosmetics and more. Thus, recombinant collagen and its preparation methodologies rooted in genetically engineered cells mark pivotal milestones in medical product research. This article provides a comprehensive overview of the current genetic engineering technologies and methods used in the production of recombinant collagen, as well as the conventional production process and quality control detection methods for this material. Furthermore, the discussion extends to foresee the strides in physical transfection and magnetic control sorting studies, envisioning an enhanced preparation of recombinant collagen-seeded cells to further fuel recombinant collagen production.

## Graphical Abstract

**Figure:**
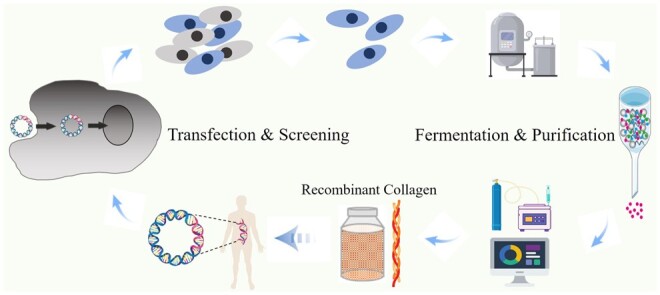

## Introduction

Collagen, the main component of the extracellular matrix, is widely distributed in human tissues, sustains the normal physiological functions of cells, tissues and organs and also plays an important role in damage repair and other processes. According to its primary structure and amino acid chain combination, different advanced structures are formed, giving rise to different types of collagen with diverse properties. To date, 29 different subtypes of collagen are known to exist in the human body [1]. Due to its excellent biological properties, collagen is widely used in the medical and cosmetics fields, manifesting as skin repair materials, bone repair materials, tendon repair materials, hemostatic materials, tissue engineering scaffold materials and drug sustained release materials [2–8]. However, the utilization of animal-derived collagen faces several challenges: risks associated with animal-borne diseases, inconsistency across batches, environmental concerns during the extraction phase and complications in eliminating protein impurities. In light of these drawbacks, there is a growing inclination towards gene recombination technology and microbial fermentation for collagen production, thanks to advances in bioengineering. The recombinant collagen prepared by this method exhibits lots of advantages, such as a single component, noviral infection, stable production process, low immunogenicity, good processability and excellent water solubility, which would broaden the horizons of recombinant collage applications [9–11]

Biologically active collagen is prepared by introducing its target genes into specific host cells, followed by gene expression and protein translation, and subsequent extraction and purification. The target recombinant collagen genes were obtained by the following steps: first, recombinant DNA technology was used to modify the gene of the human collagen coding region; second, the mRNA of collagen molecules was reverse transcribed into the corresponding cDNA; third, the cDNA was integrated into the plasmid or other carriers; and lastly, the plasmid was used to transfer the target gene. The production of recombinant collagen involves many steps, including selecting host cells, acquiring target genes, constructing recombinant plasmids, transfecting host cells, selecting positive cells, constructing expression systems, fermentation of cells, purifying recombinant collagen and so on. In this article, we review the methods of characterizing and identifying recombinant collagen, constructing a recombinant collagen engineering cell bank, discussing advancements in large-scale recombinant collagen production and prospecting emerging gene transfection and cell sorting technologies for the production of recombinant collagen.

## Construction of recombinant collagen-engineered cell bank

**Figure 1.**
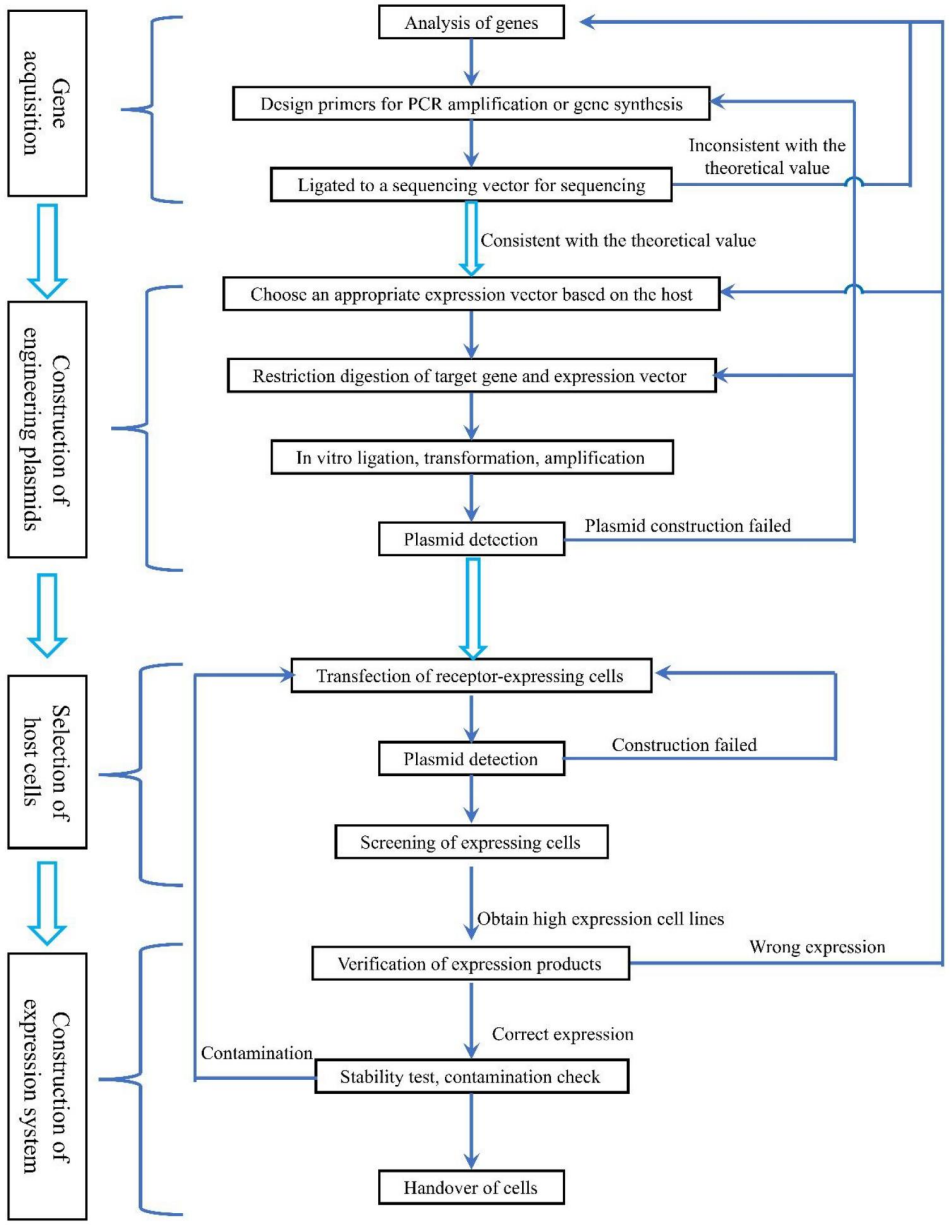
Construction process of engineered cells [12].

With the development and application of recombinant DNA technology, recombinant collagen has been expressed in different systems, including various cells (such as HT 1080 cells, CHO cells and HEK293 cells), microorganisms (such as yeast and bacteria) and transgenic animals and plants (such as tobacco, barley and corn plant cells). Central to the production of recombinant collagen is the creation of engineered cells. The construction of engineered cells includes the following stages: acquisition of target genes, construction of plasmids, selection of host cells and construction of expression systems. Figure 1 illustrates the process of the construction of engineered cells.

### Source of host cells

Bacterial, yeast and insect cell expression systems have been extensively investigated and developed due to their ease of industrial-scale production and expression. In the actual production of engineered recombinant collagen, the host cells chosen for constructing engineered recombinant cells must have traceable origins and a clear culture history.

When selecting a host system for the expression of recombinant collagen, a comprehensive understanding of its characteristics is crucial. The characteristics of the host system (prokaryotic, eukaryotic, mammalian cells, etc.), genetic background, genotype, phenotype, resistance and characteristics of plasmid transformation conditions are generally available in detail. For clarity, Table 1 lists the characteristics of the three host cells commonly used in the production of recombinant collagen from prokaryotic, yeast and animal cells.

**Table 1.** Basic characteristics of typical host cells

Name of cells	Chinese hamster ovary (CHO) [13]	*Pichia pastoris* [14]	*Escherichia coli* [15]
Company	Sigma	Invitrogen	Invitrogen
Type of host system	Chinese hamster ovary cells	*Pichia pastoris* GS115	*Escherichia coli* BL21(DE3)
Growth conditions	36.5°C with oxygen	28°C with oxygen	37°C with oxygen
Transfection method	Electroporation	Electroporation	Electroporation
Genotype	Wild type	His4	F-ompT hsdSB(rB-mB-)gal dcm rne131(DE3)

As the host cell, its contamination with microbes and auxotrophic phenotype (or gene knockout), as well as overexpression and introduction of foreign genes, should be detected. The detection items and methods are shown in Table 2.

**Table 2.** Typical host cell identification items [16, 17]

Check item	Method	Quality control
Contamination	Plate streaking, microscope detection, mycoplasma detection kit	Uncontaminated
Auxotrophic phenotype identification	Auxotrophic plate growth identification	Not growing
The availability of methanol	Methanol-containing plate growth identification	Consistent with strain phenotype
Identification of imported foreign genes	Polymerase chain reaction (PCR) amplification, product sequencing	Compatible with the genotype of the strain
Identification of Overexpressed Genes	Quantitative PCR (qPCR) detection of transcript levels of overexpressed genes	Consistent with strain phenotype

### Acquisition of the target gene

The literature and public databases, including the National Center for Biotechnology Information (NCBI) bioinformatics database (http://www.ncbi.nlm.nih.gov/), are the commonly used database to acquire the amino acid sequence of human collagen and its corresponding nucleic acid sequence, which encompassed the complete human amino acid sequence, fragment human amino acid sequence and fragment repeats human amino acid sequence. Then, according to the information from the bioinformatics database, the target fragment of the human amino acid sequence (gene sequence) was selected. Methods to obtain target genes include isolation from naturally occurring genes, gene library isolation, PCR separation and artificial synthesis. Direct isolation from natural genes and gene library isolation are suitable for obtaining unknown genes and the PCR separation method and artificial synthesis are used to obtain known genes. The direct isolation method is user-friendly and straightforward; however, it bears a significant limitation: the randomness of the procedure often makes isolating the target gene challenging. Gene library isolation is a simple method to obtain target genes. When using a gene library to obtain a target gene, information related to the target gene such as nucleotide sequence, gene function, position on the chromosome and transcripts should be considered. PCR amplification utilizes the thermal denaturation characteristics of DNA to open the DNA strands under certain temperatures, lower the temperature for primers to attach the DNA template and then extend under certain temperature conditions to obtain the target gene [18]. Artificial synthesis generally includes three methods, namely, the small piece bonding method, patch extension method and large fragment enzymatic method. The principle of chemically synthesized DNA is to connect individual deoxynucleotides in the required order, and each monomer is connected in a PCR cycle [19].

The correct selection of the target gene determines the formation of the triple helix region (collagen domain) in the recombinant collagen molecule. The following items (in Table 3) should be considered when measuring the quality of the target gene.

**Table 3.** Typical items to measure the quality of target genes [20]

Check item	Method	Quality control
Target gene sequence	DNA sequencing	Correct sequencing result
Accuracy of restriction enzymes	electrophoresis of Nucleic acid	The insert size is correct and there are no unexpected fragments
Quality of DNA	UV scanning	OD260/280 = 1.8∼2.0

### Construction of recombinant plasmids

**Figure 2.**
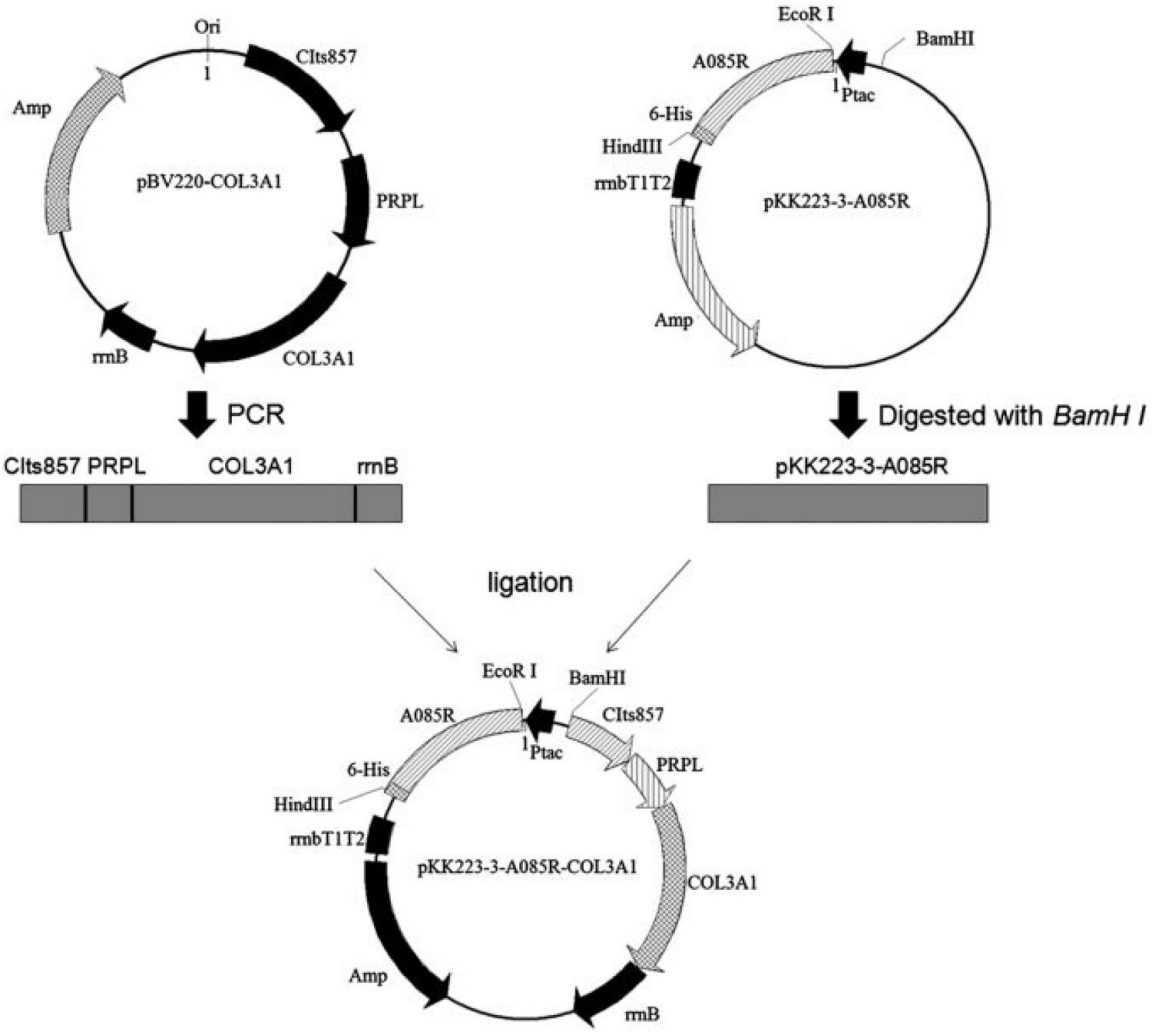
Schematic map of the coexpression vector pKK223-3-A085R-COL3A1 construction. The prolyl 4-hydroxylase gene (A085R) was derived from the PBCV-1 genome, and then the DNA product corresponding to nucleotides 36–242 of the A085R open reading frame was amplified by PCR. pKK223-3-A085R (A085R NCBI reference sequence: NP_048433.1) was linearized with B*amH*I. And pBV220-COL3A1-6His (COL3A1cds GenBank: BC028178.1) was constructed by PCR. Then, the purified PCR fragment CIts857-PRPL-COL3A1-rrnB and the linearized vector pKK223-3-A085R were connected using reorganization Kits. CIts857, expression suppressor gene, regulated by temperature; PRPL, tandem promoter; rrnB or rrnbT1T2, terminator; Amp, ampicillin; Ori, origin [23].

Similar to the gene recombination process of other biological proteins, after the (Gly-X-Y)_n_ target gene of collagen is obtained, the target gene is combined with the carrier (plasmid) to construct the target gene expression vector. The construction process of the plasmid vector includes cutting the plasmid and the target gene with restriction enzymes, exposing the same cohesive ends or blunt ends and DNA ligase connecting the ends to form target recombinant DNA molecules through DNA chain ligation [21]. In the process of constructing the collagen target gene, it is sometimes necessary to knock out certain specific genes of the host cells to optimize the collagen expression system. Fan *et al.* [22] constructed a human collagen gene into *Escherichia coli* with the PstG gene knocked out to obtain a high-expression human-like collagen expression system. Figure 2 shows an example of plasmid construction.

For the construction of a plasmid incorporating target genes, the choice of plasmid varies based on the host cells in consideration. Complete sequence information of commercial plasmids for commercial plasmids is readily available, which generally includes details about the source and function of expression vector components and main elements, such as restriction sites, resistance markers, promoters, replicons and enhancers, the sequence of the flanking sequence region, the inserted foreign gene, etc. Furthermore, the method employed to evaluate the quality of the constructed plasmid aligns with the technique utilized to acquire the target gene (Table 3) [20].

### Transfection of host cells

**Figure 3.**
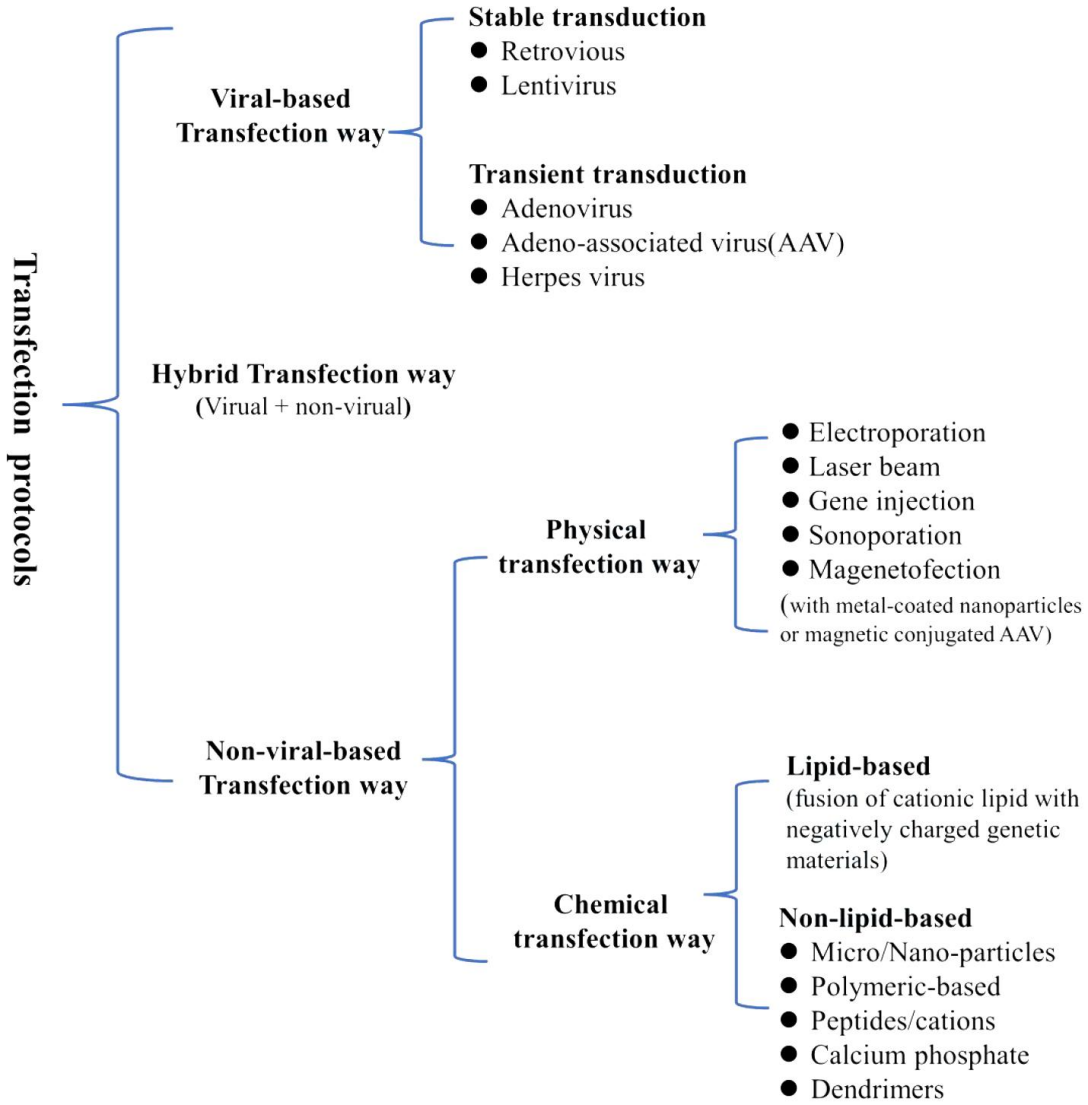
Transfection routes can also be classified as viral transfection, nonviral transfection or hybrid transfection [25].

Genetic engineering of cells involves introducing exogenous genes into host cells, using host cells to express target genes and then obtaining the target proteins. With the development of genetic engineering and molecular cloning technology, transfection has emerged as a fundamental procedure frequently deployed in cell-based research. Common transfection methods include physical transfection, chemical transfection and biological transfection. Among them, common methods for physical transfection include microinjection, electroporation, the gene gun method, etc.; chemical methods include the calcium phosphate coprecipitation method, liposome transfection method, cation-mediated method, etc.; and the most commonly used method for biological transfection includes the virus-mediated method and protoplast transfection, among others [24]. Transfection methods can also be divided into viral transfection, nonviral transfection and hybrid transfection methods according to whether viruses are used as plasmid vectors, as shown in Figure 3.

According to the length of time that the transfected target gene remains in the host cell, cell transfection methods can be divided into transient transfection and stable transfection. In transient transfection, the exogenous gene does not integrate into the nucleus or nucleoid of the host cell. As a result, it is easily lost during cell reproduction, precluding long-term stable gene expression. In contrast, stable transfection facilitates the integration of exogenous genes with the chromosomes in the nucleus which is not lost during the inheritance process, allowing for stable long-term expression of genes and proteins. However, the expression level of stably transfected genes is generally lower than that of transiently transfected genes. The transfection of host cells for recombinant collagen frequently employs the three ways listed below.

#### Viral transfection

Viral transfection speed is fast, and the transfection efficiency is extremely high, reaching 100%. Among the available systems, the baculovirus expression vector system is among the most ideal systems for the routine production and display of recombinant collagen in insect, larval and mammalian cells [26]. For the construction of plant expression systems for recombinant collagen, *Agrobacterium tumefaciens* is the most widely used transfection vector. The typical approach involves introducing the recombinant target protein genes (specifically collagen and P4H) into *A. tumefaciens* using electroporation or chemical transfection agents. Subsequently, the *A. tumefaciens* transfected with the target gene is co-cultivated with plant embryonic cells [27].

#### Physical transfection

Physical transfection is an efficient non-viral transfection method. Heat treatment has been found to enhance the total uptake of plasmid DNA and increase caveolar endocytosis. Since caveolar endocytosis is advantageous for escaping lysosomal digestion, the amount of intact DNA may increase, resulting in enhanced gene expression [27, 28]. The heat-shock method, in which cells are exposed to elevated temperatures, is a commonly used method for plasmid transfection into *E. coli*. At 42°C, *E. coli* undergoes heat shock, allowing the plasmid to enter the cell via gaps formed on the cell membrane of *E. coli*, thereby achieving transfection. Using this approach, Shi *et al.* [23] transfected a recombinant plasmid vector expressing human collagen α1(III) and hydroxylase into *E. coli* by heat-shock treatment at 42°C.

**Figure 4.**
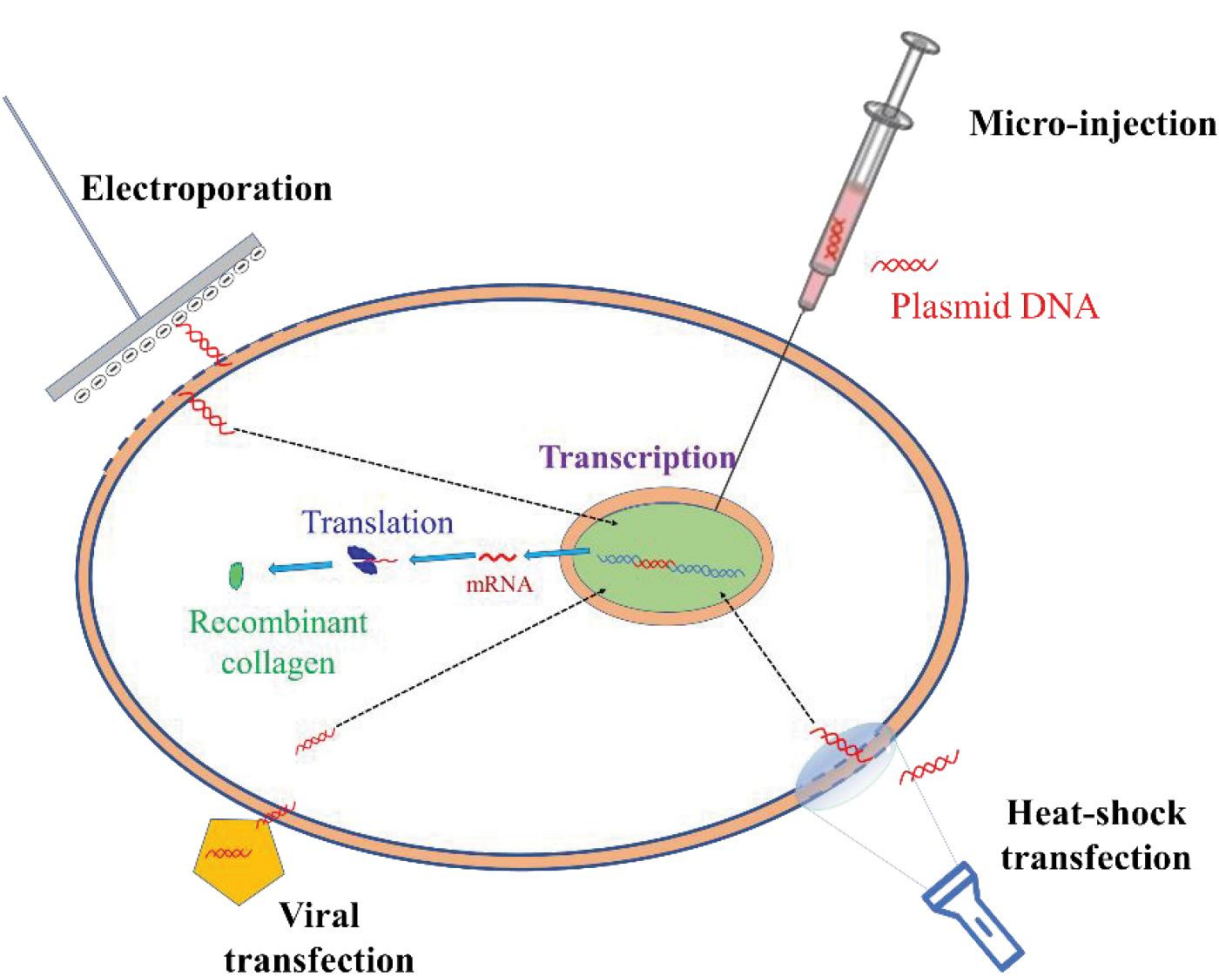
Schematic diagram of commonly used transfection methods for transfected target genes of recombinant collagen.

In addition, electroporation is another prevalent physical transfection method. This method relies on utilizing electric current to break down the cell membrane, thereby forming temporary small holes or water channels. Consequently, DNA molecules can enter the cell through these channels or membrane holes. Electroporation is generally applicable when liposome transfection is unsuccessful or the transfection efficiency is extremely low. However, it also suffers from drawbacks, in general, a high electric field strength results in the death of 50–70% of the cells. Fortunately, electroporation protection agents can effectively reduce the cell death rate while simultaneously improving transfection efficiency. Despite its potential, this method has not been used on a large scale for the transfection of recombinant collagen expression systems due to several technical deficiencies that have not been fully resolved. However, with the rapid development of micro-nano processing technology, injection transfection of host cells through micro–nano needles has become an emerging method, which will be discussed in the outlook section. A schematic diagram of different physical transfection and viral transfection methods is shown in Figure 4.

#### Chemical transfection

Chemical transfection is a commonly used method for plasmid transfection. Calcium phosphate precipitation is the most used chemical transfection method for transfecting recombinant collagen plasmids. Calcium phosphate coprecipitation transfection involves mixing nucleotides, calcium and phosphate buffer to form a precipitate that is taken up by cells through endocytosis [29]. A fraction of the nucleotides that escape the endosome can then be expressed in the target cell. However, the transfection efficiency of the calcium phosphate coprecipitation transfection method is strongly dependent on cell composition, pH and the quality and quantity of nucleotides. It is important to note that the calcium phosphate coprecipitation transfection method can be toxic, rendering it unsuitable for many sensitive primary cell lines. In a relevant study, Geddis and Prockop [30] used this method to transfect the human COL1A1 gene into fibrosarcoma cells (HT1080) to establish a stably transfected cell line. Similarly, Yamada *et al.* transfected murine α1(IV) and α2(IV) into CHO cells [31].

**Figure 5.**
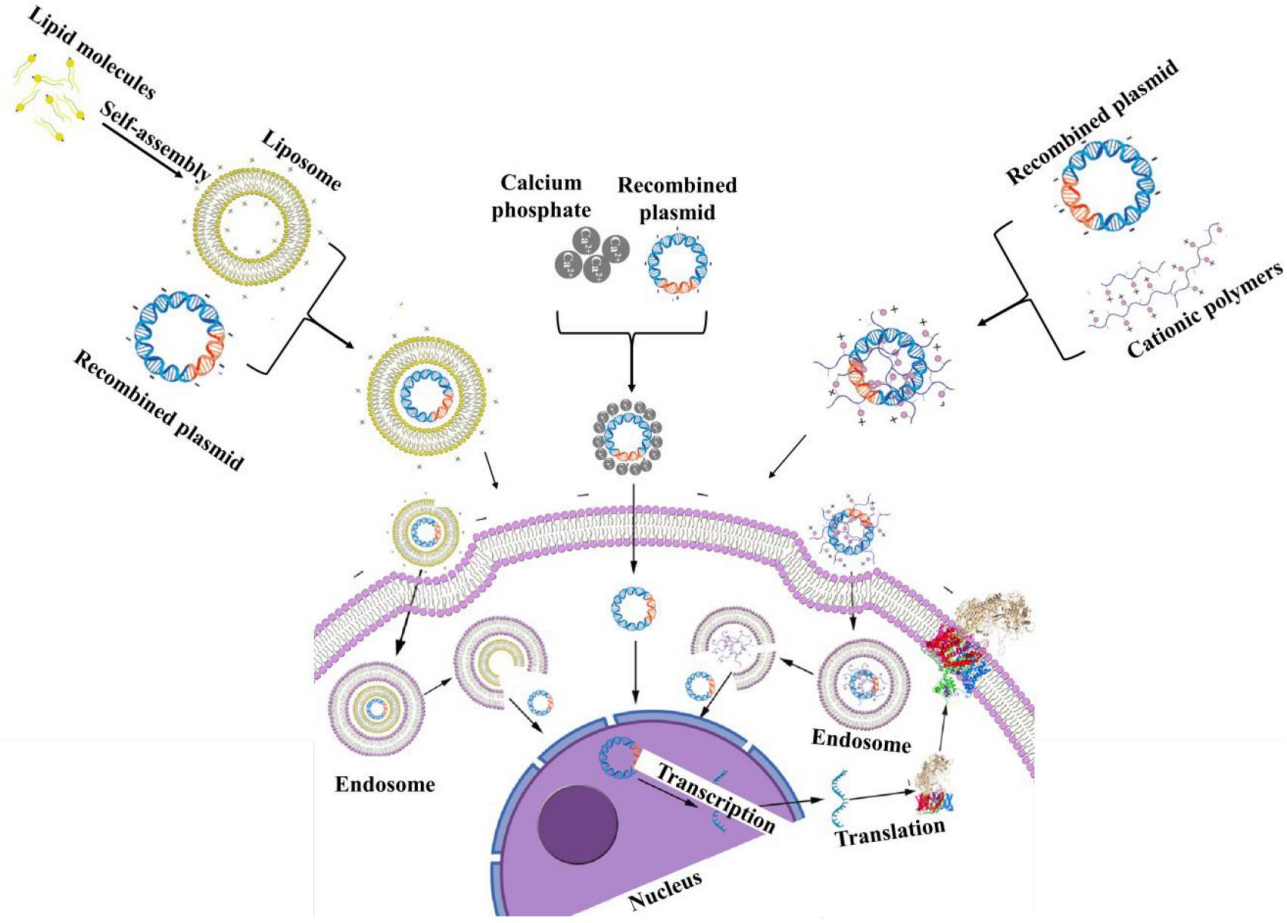
Schematic of liposome-mediated transfection, calcium phosphate coprecipitation and cationic polymer transfection methods.

Similar to the principle of calcium phosphate precipitation transfection, there are some similar transfection chemicals, such as cationic polymer transfection agents and liposome-mediated transfection agents. Cationic polymers can interact with plasmid DNA through electrostatic interaction, which results in plasmid DNA complexation and condensation. The formed positively charged complexes readily associate with the cell membrane, which increases the transfection efficiency. On the other hand, liposome-mediated transfection relies on the electrostatic binding or adsorption between positively charged liposomes and negatively charged DNA molecules or cell membranes. These complexes are then introduced into cells via endocytosis or fusion, facilitating the transfection of expression vectors. Notably, this method is especially suitable for DNA transfection of both suspensions and adherent culture cells, such as human embryonal kidney cell line 293 [32], mouse fibroblasts [33] and CHO [31] for recombinant collagen production. The method exhibits several advantages, including simple operation and high transfection efficiency. Considering the cytotoxicity of liposomes, the transfection time is generally controlled within 24 h. Although cationic polymers and liposome-mediated transfection have not yet been used for large-scale plasmid transfection of recombinant collagen host cells due to the problem of economic cost and transfection efficiency concerns, these two transfection methods hold considerable promise in transfection of recombinant collagen plasmids. A schematic diagram of the liposome-mediated transfection method, calcium phosphate coprecipitation and cationic polymers transfect methods are shown in Figure 5.

Diverse expression systems adapt their selection of transfection vectors and methods based on their intrinsic properties, with the primary goal of constructing high-expression transformants and ensuring swift and effective transfection. For example, *E. coli* expression systems are often transfected by heat shock, while yeast expression systems are often transfected by calcium phosphate coprecipitation. Human cell lines are usually transfected by electroporation, and the insect cell systems are usually transfected by virus transfection, and plant expression system leverages transfected with *A. tumefaciens* carrying the target gene. Different transfection methods exhibit corresponding advantages and disadvantages for different host cell systems, which are shown in Table 4 [34].

**Table 4.** Advantages and disadvantages of commonly used transfection methods [34]

Method	Cell type	Effectiveness	Cost	Introduced molecule	Advantage	Disadvantage
Instrument-based methods of transfection						
Microinjection	Any *in vitro* cell	Close to 100%; dependent on injected material	>$1000	Any (DNA RNA, spermatozoids, proteins, peptides. drugs.)	High efficiency; Precise dosing of injected material; selective delivery; low cytotoxicity	Maximum of 100–200 cells transfected in a single treatment; Laborious process
			Grows with process automatization level			
Biolistic transfection	*In vitro* and *in vivo*; e.g. primary leukocytes-lymphocytes. macrophages, and splenocytes	High	High cost of necessary equipment; low cost of utilization	DNA	Possible transfection through physical barriers like epidermis; possible co-transfection of more than one DNA in a single use; time efficient	High cost of gene gun; Tissue damage when transfecting small cells
				RNA		
Electroporation	*In vitro* and *in vivo*; see above	Low to moderate	>$1000	Plasmids:	High efficiency; Proven efficiency for use on tissues *in vivo*	High toxicity
				Oligonucleotides; mRNA; SiRNA		
Optical transfection	*In vitro* cells	Comparable to other physical methods	High cost of necessary equipment	DNA, RNA, and larger objects	Ability to transfect single cells; Possible transfection with large objects	Diverse efficiency depending on the technique
Virus-based methods of transfection						
Adenoviruses	Dividing and non-dividing cells	Expression levels are very high at the beginning, but they quickly weaken in a matter of weeks	$500–$1000	DNA	No integration with the host cell chromosome; Easy viruses amplification: vectors stability in prolonged storage	Cannot induce prolonged expression; tendency to induce a strong host immune response; Use possible only in laboratories with Biosafety Level 2 or higher
Adeno-associate virus	See above	See above	$500–$1000	DNA	No integration with host genome: weaker immunogenicity than adenovirus	Cannot induce prolonged expression
Retroviruses	Dividing and non-dividing cells	Stable expression	≈$1000	RNA	Stable transfected gene expression	Possible retroviral genotoxicity
Chemical transfection methods						
Calcium phosphate	*In vitro* cells	High	<1000$	DNA	Inexpensive; high efficiency: applicable to a wide range of cell types; allows transient and stable transfection	transfection efficiency is influenced by small changes in pH; consistency of precipitate
Cationic lipids	*In vitro* and *in vivo*	High	<1000$	DNA, RNA, siRNA, and proteins	high efficiency: easy procedure: DNA RNA and proteins may be introduced: allow for transient and stable transfection	does not work with certain cell types
DEAE-Dextran	*In vitro* cells	Moderate	<1,000$	DNA and RNA	inexpensive; quick and easy method; wide range of cell types may be transfected: DNA and RNA may be introduced	toxicity of DEAE-dextran high concentrations; only for transient transfection: proteins may not be introduced
Magnetic beads	*In vitro* cells	High	<1000$	DNA and RNA	simple method; high efficiency: DNA and RNA may be introduced	only adherent cells may be transfected; cells in suspension must be immobilized

### Screening for positive cells

**Figure 6.**
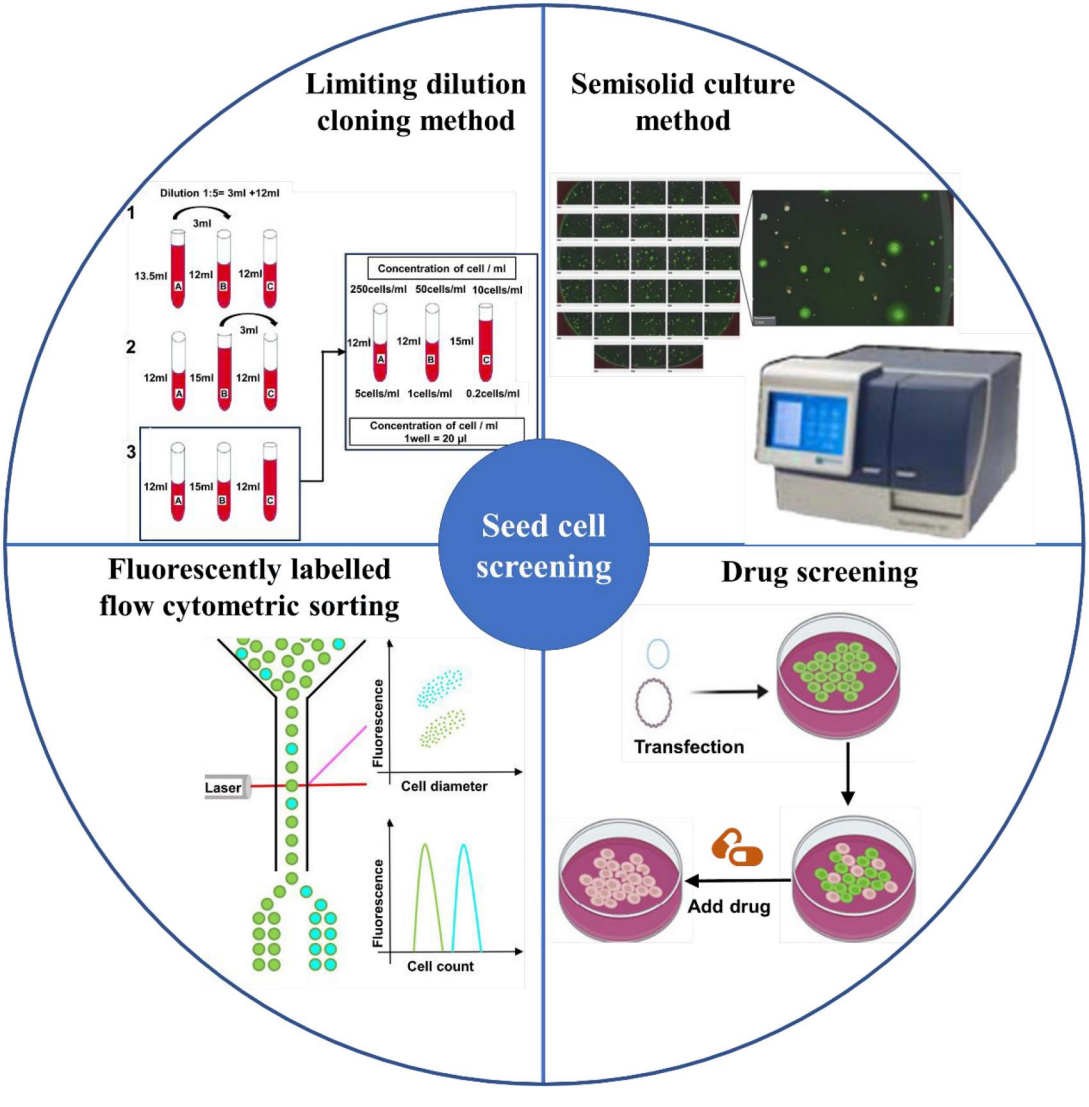
The main methods for seed cell sorting.

After the plasmids of target genes are transfected into the host cells, it is very important to screen for the positive cells. These chosen positive cells are characterized by their proficiency to stably express the target protein, ensuring that it retains its structural integrity and biological activity. This is of paramount importance in the establishment of a cell bank that exemplifies robust growth potential, superior product quality and consistent heritability. Commonly used screening methods are shown in Figure 6, including the limiting dilution cloning (LDC) method, semisolid medium method, flow cytometry sorting method and drug screening method. Various screening methods are introduced in detail in the following sections.

**Figure 7.**
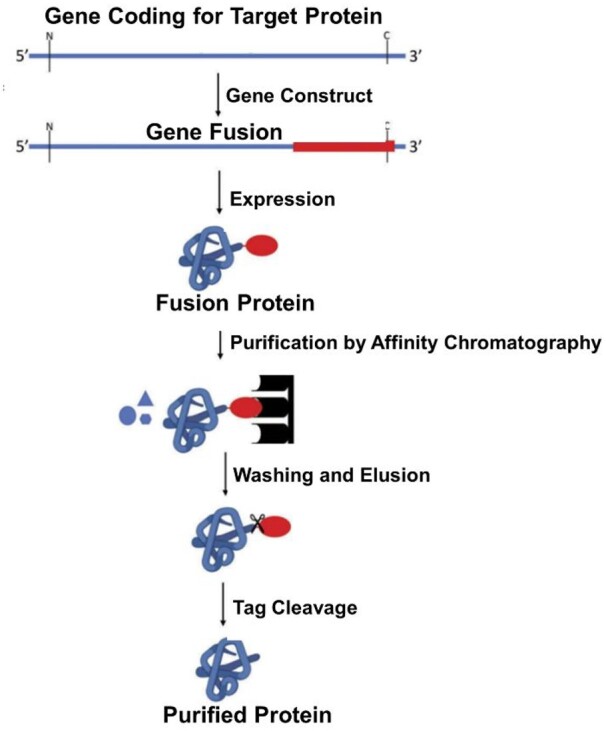
Steps involved in purification of recombinant collagen by affinity chromatography [72].

#### LDC method

LDC is a method used to obtain monoclonal cells by serially diluting cells through inoculation, screening and amplification. LDC is widely used because it is inexpensive and easy to operate. For a broad spectrum of cell types, such as stem cells and hybridoma cells, LCD facilitates the isolation of monoclonal cells. The operation procedure initiates with serial dilution of the inoculated and cultured cell solution with cell culture medium and then the diluted solution is inoculated into a 96-well plate. Given the extent of dilution, theoretically, each well should house no more than a single cell. Although LCD offers precision, this method is laborious, requires a significant amount of time and effort and often requires the use of automated workstations for high-throughput screening.

#### Semisolid culture method

The semisolid culture method is used to achieve cell dispersion, propagation and formation of individual cell colonies in an environment with low fluidity. The development of automated processes and microscopic imaging technology has greatly improved the selectivity, effectiveness and flexibility of this method. With the formation of monoclonal cell colonies, the target protein is secreted and expressed and specifically binds the fluorescently labeled secondary antibody in the medium. Through capturing and fluorescent imaging, cell colonies with high expression levels are screened out, and then cells that meet the requirements are selected by an automated robotic arm. A noteworthy application comes from Hou *et al.* who employed the ClonePix FL system, developed by Genetix, for selecting target clones. This selection was grounded on parameters like fluorescence value, size and morphological irregularities, using combined analyses of fluorescence and white light imaging [35]. In the study, cells were plated at 1000 cells/well in a semisolid medium and analyzed using a ClonePixTM FL. A singular well with a glass base was employed for imaging, divided into 28 distinct sections. mAb expression was evident through the accumulation of a green-tinted fluorescent detection reagent that interacted with the secreted mAb. The ClonePixTM FL software allowed for a comprehensive assessment of each colony using user-specified parameters, encompassing aspects such as size, morphology, fluorescence and positioning [35].

#### Fluorescently labeled flow cytometric sorting

In the fluorescent flow cytometry sorting method, the fluorescent secondary antibody of the target protein is used to screen the transfected cells. Specifically, the fluorescence intensity is indicative of the cells that highly secrete the target protein. This approach provides a quantitative measure of protein expression level. To ensure optimal binding of the fluorescent secondary antibody and the target protein, it is also necessary to encapsulate the cell and the secreted target protein with microcapsules to keep the target protein on the cell membrane surface. Improving and upgrading detection and operation methods is very important for clone screening, accommodating microvolume and achieving high throughput; for example, using homogeneous time-resolved fluorescence with high-throughput and a small sample volume (2.5–5.0 μl) (384-well plate) detection. The integration of this technique with automated workstations can substantially alleviate the research workload. Fluorescently labeled cell sorting is a reliable and safe method to obtain pure vital monoclonal cell lines from mixed human cells with activity to form new tissues [36]. In a previous study, Mori *et al.* successfully identified cell lines that highly express the target protein by fluorescence intensity with the help of green fluorescent protein (GFP) fluorescent labeling [37].

#### Antibiotic screening

The principle that recipient bacteria without antibiotic resistance genes cannot grow in a medium containing antibiotics can be used to screen transformants with high expression of the target gene. Then, the transformants are verified and confirmed by gel electrophoresis or PCR. Because most plasmids are marked with antibiotic resistance, antibiotic selection is the most widely used method for selecting positive cells. Commonly used antibiotics include G418, ampicillin and kanamycin. In a notable endeavor, Geddis and Prockop introduced the human COL1A1 gene into fibrosarcoma cells (HT1080) to construct a stably transfected cell line. Two days after the transfection, the cells were relocated to a culture dish where G418 was employed as a selective agent to discern the transfected entities. Finally, cell clones with G418 resistance were collected for verification and confirmation, and cell lines with high expression of the target gene were screened out [30].

Integrative approaches combining both antibiotic screening and fluorescence sorting have been documented. Wieczorek *et al.* [38] co-transfected the telopeptide human COL2A1 gene and enhanced cyan fluorescent protein into HT1080 human fibrosarcoma cells. Selective antibiotics (G418, Mediatech) were added to the culture system to eliminate cells with unstable expression, and then, the cyan fluorescence was detected to screen and amplify cells with highly stable expression.

### Construction of the expression system

To construct the expression system of the target protein, a target gene is combined with the vector and transformed into the host cells. The expression systems of recombinant collagen generally fall into the following categories: microbial expression systems, including *E. coli* (prokaryotic) and yeast (eukaryotic) expression systems, animal and plant expression systems [39–41].

#### *E. coli* expression system

*E. coli* currently stands as the foremost prokaryotic cell expression system utilized in the production of recombinant proteins. It is responsible for ∼40% of the clinically used recombinant proteins, amounting to over 400 varieties [42–45]. Its advantages include. A well-defined genetic background, an intricate regulatory mechanism, an exhaustive range of commercial vectors and strains, cost-effectiveness, a short growth cycle and exceptional expression efficiency. Rutschmann *et al.* [46] constructed an *E. coli* expression system capable of simultaneously expressing proline hydroxylase and human type III collagen. The obtained hydroxylated human recombinant type III collagen has good biocompatibility, and experiments have shown that it can promote the growth of umbilical endothelial cells. There are also some obvious deficiencies associated with the *E. coli* expression system, such as the inability to glycosylate proteins, low biological activity, formation of insoluble inclusion bodies due to insufficient secretion capacity and the arduous task of eradicating endotoxins it generates [47]. Enormous efforts have been devoted to solving these issues [23, 48].

#### Yeast expression system

The yeast expression system exhibits several advantages, including the absence of endotoxin, the ability for large-scale high-density culture, simplicity in operation and cost-effectiveness. The system has been extensively studied and industrialized [47]. Owing to the rich diversity and distinct attributes of yeast, the choice of host strain should be tailored to the specificities of the target gene. Commonly used yeast expression types include the following: methanotrophic yeast, the most prevalently adopted yeast, requires methanol as its carbon source and the methanol concentration directly impacts its expression level; *Saccharomyces cerevisiae*, the earliest yeast expression system for a long history, the foreign genes of which are easily lost and the expression level cannot keep up with the demand; *Kluvia lactis*, with high safety and protein expression levels, can be used for large-scale industrial production of protein and *Pichia pastoris* is among the most widely used strains for constructing a recombinant collagen expression system; *Yarrowia lipolytica*, which can secrete many proteins with a low degree of glycosylation; and *Schizosaccharomyces pombe*, which can express many products similar to natural proteins, is conducive to large-scale application.

#### Animal and plant expression systems

Commonly used host cells for animal expression systems include insect cells, mammalian mouse cells and silkworms. The advantage of the insect expression system is that the recombinant collagen retains the complete protein structure and biological activity, can accept the insertion of large fragments of foreign genes and enables extracellular protein expression. The disadvantages are also obvious, including glycosylation that is different from that of mammals, limited protein expression and high cost. The protein expressed by the mammalian expression system is very similar to the human protein and has a complete structure, and appropriate posttranslational modification (glycosylation, hydroxylation, etc.), leading to high biological activity. However, transitioning to large-scale production proves daunting, primarily due to financial constraints. On the other hand, when plants are utilized as hosts for collagen expression, tobacco and corn cells emerge as primary candidates. They offer the advantages of no risk related to animal pathogen infections and the ease of foreign gene integration. The advantages and disadvantages of different expression systems are summarized in Table 5. Different expression systems have been used to produce recombinant collagen, and the expression systems used for recombinant collagen are reviewed and summarized in Table 6.

**Table 5.** Advantages and disadvantages of different expression systems [49]

Expression system	Advantages	Disadvantages
Microbes	Clear genetic background; strong operability; low cost; suitable for large-scale production.	For the *Pichia pastoris* system, methanol is a potential risk; *Escherichia coli* system, difficult to purify, low product activity.
Animals	Suitable for the expression of complete macromolecular proteins, which can be secreted and expressed; High protein activity, correct advanced structure and protein modification (such as hydroxylation, glycosylation).	High technical requirements; high culture costs; long time-consuming; low expression; not conducive to industrialization.
Plants	Wide range of host sources and low cost; high security.	Long growth cycle; high cost of fermentation and purification; low yield; not conducive to industrial production.

**Table 6.** Summary of expression systems for recombinant collagen production (updated on [50])

Expression system	Examples of collagen constructs	Requirement for co-expression of P4H	Industrial-scale production	Commercial evaluation
		(N = no, Y = yes)		
Mammalian cells (HT1080, CHO, KEK293, NIH3T3)	Native-like human procollagens, including procollagen I, procollagen II, collagen VI, procollagen VII, fragments of procollagens, including mini-collagen II, mini-collagen I homotrimer, mini-collagen VII, C-terminal propeptides of procollagen III, and fragments of collagen IV	N	N	N
Insect cells	Native-like collagens including collagen I, collagen II, collagen III, collagen IX, collagen	N	N	N
Mammary gland of transgenic mice	Collagen I homotrimer	N	N	N
*Escherichia coli*	Human-derived mini-collagen III, collagen fragments, including C-propeptide collagen XVIII, and fragments of collagen I	Y	Y	Y
*Escherichia coli*	Collagen fragments stabilized by bacterial collagen-like sequences	N	N	N
Yeast cells	Native-like human collagen I, collagen III, gelatin	Y	Y	Y
Transgenic plants	Native-like human collagen I	Y	Y	Y

The hallmark of a successfully constructed expression system is the engineered cells' ability to stably and efficiently express the target product with both structural correctness and biological activity. Tables 7 and 8 provide examples of evaluation and analysis of the success of engineering cell construction and the stability of target protein expression, respectively.

**Table 7.** Evaluation of engineered cells [51]

Evaluation items	Method	Evaluation criteria
Linearization of engineered plasmids	Nucleic acid electrophoresis	Single strap, correct size
Purification of linearized plasmids	UV spectrophotometric scanning	DNA concentration not lower than 100 ng/μl: OD260/280 = 1.8∼2.0
Transfection of host cells	Plate screening, PCR amplification product sequencing,	Correct sequence
Identification of expression proteins	SDS‒PAGE electrophoresis Coomassie Brilliant blue staining	The molecular weight as expected
	Western blotting	The molecular weight as expected
	Hydrolyzed amino acid detection	Correct amino acid composition
Screening of expression	SDS‒PAGE/Western blotting/ELISA (except yeast)	Selected according to the top 5% of expression

**Table 8.** Stability analysis of target protein expression [52]

Evaluation items	Method	Evaluation criteria
Stability of expression	SDS‒PAGE electrophoresis Coomassie Brilliant blue staining	Consistent with the standard
Contents	UV spectrophotometric scanning	Not lower than the initial engineering bacteria
Contamination detection	Microscopic detection, mycoplasma detection kit	No contamination

## The production process of recombinant human collagen

The high cost of animal and plant expression systems limits their industrial transformation; On the other hand, the microbial system emerges as the best option for large-scale and cost-effective production of recombinant human collagen [53–55]. To achieve high-yield, high-purity and high-stability expression systems, it is necessary to screen for suitable host cells, gene carriers and culture conditions. Notably, the expression and purification processes for prokaryotic and eukaryotic microbial systems are quite different from each other. The details are shown in Table 9.

**Table 9.** Comparison of the expression and purification process for prokaryotic and eukaryotic microbial systems

Microbial systems	Expression	Purification
Prokaryotic	Affected by pH, temperature, dissolved oxygen, acetic acid, carbon source, nitrogen source, etc.	Mostly expressed intracellularly and intracellular substances need to be released first. Involved precipitation, ultrafiltration, chromatography and other steps.
Eukaryotic	Some require methanol induction, affected by pH, temperature, methanol content, etc.	Both endocrine and exocrine are possible, and difficult to disrupt cells when released intracellularly, involving precipitation, ultrafiltration, chromatography and other steps.

### Fermentation process

#### Fermentation of prokaryotic cells

*E. coli* is a commonly used prokaryotic expression system, and the fermentation process of *E. coli* is influenced by multiple factors, including carbon and nitrogen concentration, dissolved oxygen, temperature, pH and others. An appropriate basal carbon and nitrogen concentration is indispensable during the cultivation process. A nitrogen concentration that is too high will lead to the vigorous growth of the bacteria, leading to premature aging and autolysis. Conversely, an overly high carbon concentration can cause a metabolic imbalance in bacteria, resulting in limited bacterial reproduction, which is not conducive to the accumulation of target protein. In addition, Insufficient carbon and nitrogen levels do not favor bacterial biomass increase, making it challenging to attain higher fermentation density [56]. For example, Zhang *et al.* constructed recombinant human type III collagen in *E. coli*, optimizing the carbon and nitrogen concentration of the medium through orthogonal experiments. The results showed that the optimum formulation was peptone at 15 g/l, yeast powder at 7.5 g/l and glycerin at 12 g/l. The bacterial count increased by ∼120%, and the expression amount reached 10.31 g/l, with an increase of 199%. The yield of purified recombinant type III humanized collagen after optimization reached 78%, with a purity higher than 95% [56]. Wei *et al.* studied the bacterial growth rate and protein expression of bacteria under different concentrations of nitrogen and carbon sources. The results showed that at a glucose concentration of 10 g/l, the recombinant engineered bacteria had a cell density of 4.5 and a protein expression level of 15.55%. Under the organic nitrogen source of peptone at a concentration of 15 g/l, the cell density reached a maximum of 4.62. With the mixed nitrogen source, the cell density and protein expression reached their highest levels of 10–15 g/l [57].

In addition to the carbon and nitrogen source, dissolved oxygen, temperature and pH also have an impact on the expression of collagen in prokaryotic systems. Wei *et al.* found that at pH 6.5, the exogenous protein content was the highest, reaching ∼33.31%. Reducing the culture temperature notably enhanced collagen expression, albeit with a slight decline in bacterial concentration. To study the impact of starvation on protein expression, Xue *et al.* prevented the accumulation of acetic acid by restricting the sugar supply at various stages. Their findings revealed that the protein concentration was lowest at 1.5 g/l sugar post-induction, followed by a drop in cell dry weight. Before induction, the dry weight of cells reached a maximum, with the lowest acetic acid content. Moreover, they also demonstrated the role of CO_2_ in the growth and production of microbial cells by injecting CO_2_ pulses in different concentrations at different cell growth stages. The results indicated that CO_2_ injection at the culture stage can significantly inhibit cell growth. Yet, a mild enhancement in growth was observed during the fed-batch culture stage [58]. Moreover, the strain of the engineered bacteria may also influence the expression of collagen. Change found that the DS-pCYFcopt strain under isopropyl β-d-1-thiogalactopyranoside (IPTG) induction of 0.05 mM achieved the highest protein expression [59].

Another important factor that influenced the expression level is IPTG induction parameters, including the amount added, its concentration and the duration of induction. Ideas regarding the function of IPTG are controversial. Wu found that the amount and concentration of IPTG had a weak influence on protein expression, while the induction time had an obvious influence; the expression of the recombinant protein was highest when the expression of the protein was induced for 0.5 h [60]. Guan *et al.* believed that all three parameters had significant effects on the expression level. The results showed the optimal collagen expression (19.3%) occurred when IPTG was added as an inducer after 2.5 h of culture (OD value 0.7). Moreover, as the concentration of IPTG increased, the expression level of the recombinant protein also increased. In addition, the expression level of the recombinant protein was the highest (20%) when the induction time was 8 h [61].

In summary, multiple factors may influence the expression level of the prokaryotic system such as *E. coli* during different stages, including but not limited to the medium composition, strain quality and size, culture temperature, pH, dissolved oxygen concentration, IPTG addition time, concentration and amount.

#### Fermentation of eukaryotic cells

The *P. pastoris* expression system is a typical eukaryotic expression system that has been researched and developed rapidly in recent years due to its simple gene expression regulation and short growth and reproduction cycle [62, 63]. *P. pastoris* harnesses the promoter of the alcohol oxidase 1 gene (AOX1) to induce protein expression in the presence of methanol. This system is tightly regulated, meaning that in the absence of methanol, the target protein is not produced, which prevents undesirable metabolic stress on the cells during the growth phase. *P. pastoris* has a strong preference for aerobic growth, facilitating easy scale-up in bioreactors for industrial applications [64]. Guo *et al.* found that the protein expression of recombinant collagen in *P. pastoris* was significantly affected by methanol content, pH and temperature. The protein expression reached the maximum theoretical value of 27.84 mg/l when the methanol addition amount was 2.13%/24 h, the pH was 4.69, and the induction temperature was 28.23°C [65]. Zhang *et al.* found that protein expression increased with increasing glycerol/methanol ratio, with a final protein expression as high as 508.46 mg/l.

Zhang *et al.*'s study shed light on the intricate balance of nutritional factors and their effects on protein expression in *P. pastoris*. It was found that 20 g/l was the optimal concentration of peptone to maximize protein concentration. Methanol is used as an inducer for the AOX1 promoter system in *P. pastoris*. However, at high concentrations, methanol can exert a toxic effect on the cells, affecting their growth and protein expression capabilities. Thus, maintaining an optimal methanol concentration in the culture medium is vital for efficient protein expression without hampering cell health. While salts are essential for yeast growth, a very high concentration can have deleterious effects. By reducing the inorganic salt concentration, the study observed an increase in the cell concentration of recombinant *Pichia*, indicating better growth conditions. Moreover, the addition of casein hydrolysate at the right time (96 h post-induction in this case) and the correct concentration (20 g/l) led to a significant increase in the secretion of recombinant collagen by ∼90% (35.85 g/l) [66].

Therefore, the yeast expression system, with its distinct advantages, still requires careful optimization to achieve maximal recombinant protein yields. More specifically, in the yeast fermentation process, the amount of methanol added plays a pivotal role in controlling the expression of the target protein. In addition, the composition of the medium, pH, casein additives, temperature and dissolved oxygen may also have extensive effects on the expression of collagen and cell density [58, 67, 68]. Therefore, the optimal expression level can be obtained by precisely regulating the fermentation conditions during the protein production process.

### Purification process

To obtain high-purity collagen, the purification process is necessary after the expression of the microbial system, which is to remove the impurities such as the cells or cell fragments, cell metabolites, endotoxins and other residual substances [69]. Given that the protein produced by the *E. coli* expression system resides intracellularly, the bacteria must be lysed to release the protein. The crushing methods include enzymatic dissolution, ultrasonic crushing and high-pressure homogenization. Among them, high-pressure homogenization with the highest crushing rate is the most widely used, which results in the highest rate of collagen release with the least time and simple post-processing [70].

Following the intracellular release of the protein through cell lysing, primary purification and refined purification are conducted. The commonly used primary purification methods include salting out, organic solvent precipitation and ultrafiltration. The salting-out method works by exposing the hydrophobic region of the protein, thus destroying the hydration layer on its surface and causing the protein to precipitate. Different proteins can be precipitated by adjusting the salinity in the solution, to achieve the purpose of separation and purification. The most commonly used salt in the salting-out method is ammonium sulfate, which has a high solubility, low cost, little negative influence on the biological activity of the protein and an antibacterial effect at the same time [71]. Sodium chloride is another frequently used salt for precipitation. In the organic precipitation method, proteins are purified by reducing water activity using organic solvents, leading to the disruption of the protein's hydration layer and subsequent precipitation. Commonly used organic precipitants are methanol, ethanol and acetone. However, a limitation of organic precipitation is that it needs to be operated at low temperature. While ammonium sulfate as a precipitant tends to yield higher protein purity, benefiting subsequent purification stages, organic solvents often provide superior recovery rates. Besides, ultrafiltration employs a membrane's porous structure, enabling the separation of different-sized molecular materials based on pressure differences, offering simplicity and ease of operation.

After primary purification, further refinement is carried out to improve the purity of collagen. The commonly used refined purification processes include gel filtration chromatography, hydrophobic chromatography and affinity chromatography, Fig. 7 shows the an example involved in purification and recombinant collagen by affinity chromatography [72–74]. Wang *et al,* successfully separated and purified recombinant collagen from cell lysate on a large scale, employing a combination of ammonium sulfate precipitation, DEAE-52 anion exchange and Sephadex G-100 gel filtration. The recovery rate of the final product was 80.5%, and the purity reached 98%, which is suitable for industrial production [75]. Liang *et al.* adopted a one-step chromatography method to efficiently prepare recombinant human type III collagen from *P. pastoris* fermentation broth. The purification process is simple, stable and reliable, and ion exchange chromatography is easy to amplify [76] (Fig. 7).

## Characterization of recombinant collagen

The characterization of the physical and chemical properties of recombinant collagen materials requires rigorous monitoring of the preparation process, characteristics and risk control requirements. To ensure the high quality of collagen product, it is necessary to identify recombinant collagen, test the characteristics of its molecular structure and combine product characteristics (fermentation bacteria, specific performance index requirements corresponding to the species, amino acid sequence, protein spatial structure, purification process, dosage form and collagen type). In 2022, the world's first standard of recombinant collagen ‘YY/T 1849-2022 Recombinant Collagen’ was issued, documenting the quality of recombinant collagen control, testing items, stability, biological evaluation, packaging, transportation and storage. In addition, the ‘ASTM F3089-14 Standard Guide for Characterization and Standardization of Polymerizable Collagen-Based Products and Associated Collagen-Cell Interactions’ also includes some aspects of recombinant collagen.

Furthermore, to ensure the safety and effectiveness of the product, recombinant collagen should be analyzed to determine its necessary physical and chemical properties, biocompatibility, immunogenicity, purity and impurities. Various other characteristics were analyzed to affirm whether it meets the design expectations, for example, the molecular weight and isoelectric point and posttranslational modifications (deamidation, oxidation, glycan mapping/glycosylation modification and proline hydroxylation), as well as the conformation, aggregation and/or degradation state and advanced structure of the final product.

For the physical, chemical and biological properties, the corresponding national and industry standards usually make a thorough illustration, which is a guide for the technical indicators formulation of the final products. China is at the leading position in the research, development and industrialization of recombinant collagen. Therefore, the regulatory authorities of China have issued multiple regulations to control its industrialization and commercialization. For instance, ‘YY/T 1888–2023 Recombinant Humanized Collagen Protein’ specifies the quality control, technical requirements, experimental methods, stability, biological evaluation, packaging, transportation and storage, etc., of recombinant humanized collagen. ‘YY/T 1849-2022 Recombinant Collagen Protein’ lists the quality control, detection indicator and detection methods of recombinant collagen that are used as raw material for medical devices. ‘Guiding Principles for Evaluation of Recombinant Humanized Collagen Raw Materials’, which serves for registration, specifies the general requirements for recombinant humanized collagen raw materials for medical devices, applicable to all types of human collagen. All the above-mentioned guiding principles and standards have listed the requirements and common detection methods for recombinant collagen.

While for the biocompatibility and immunotoxicity of recombinant collagen, belongs to the scope of biological evaluation of medical devices. For this inspection, we can refer to the relevant requirements of GB/T 16886 for evaluation or conduct a comparative analysis with evaluation data of similar products on the market. For example, the GB/T 16886.1 specifies the path and projects for biocompatibility evaluation based on the duration time and nature of exposure, which are classified into short-term, long-term, lasting contact and surface devices, external access devices and implantable devices, respectively. While for immunotoxicity, the GB/T 16886. 20 specifies the evaluation path and methods based on the duration time and nature of exposure, which may include the inflammation, immunosuppressive response, immune stimulation, hypersensitivity reaction and self-immune reaction. These are usually evaluated by animal experiments based on the scope of usage.

### Identification of recombinant collagen

The common physiochemcial properties that should be considered of recombinant collagen include appearance, visible foreign substances, solubility, moisture content, residue on ignition, pH, dynamic viscosity, thermal stability, quantity, etc. The detailed items and commonly identified methods are listed in Table 10.

**Table 10.** Physicochemical identifications of recombinant collagen

Physiochemical items	Requirements	Identified methods
Appearance	white/yellow or transparent liquid or gels; white/like-white powder or sponges in the solid state	Visual identification
Visible foreign substance	No obvious foreign substance	Light inspection
Solubility	The solubility in water, dilute acid and neutral salt solution should be characterized and elaborated.	Visual identification
Moisture content	Meet the technical requirements	Thermogravimetry Analysis (TG)
residue on ignition	Meet the technical requirements	High temperature burning
pH	Meet the technical requirements	pH meter
Dynamic viscosity	Meet the technical requirements	Rotational viscometer method
Thermal stability	No gelation or visible floc	Differential Scanning Calorimetry (DSC)
Quantity	Meet the technical requirements based on the forms	–

As for the appearance, it should be white/yellow or transparent liquid or gels or white/white-like powder or sponges in the solid state, which can be analyzed by visual identification. The visible foreign substance and solubility could be confirmed by visual inspection, and there should be no visible foreign substance in the final products. TG measurement is a commonly applied method to characterize the moisture content of the product. The dynamic viscosity of the recombinant collagen in the gel format should be considered by the rotational viscometer method, the experimental parameters of which should be elaborated. Besides, to determine the thermal stability of the final products, DSC measurements or equivalent methods should be used to determine their stability after heating. Moreover, the quantity of the final products should be checked, where volume is the main assessment indicator for gels or liquids, while size for products of sponges and films.

For the identification of recombinant collagen, the common items that should be considered include amino acid sequence, molecular weight and isoelectric point. The isoelectric point could be identified by the isoelectric focusing electrophoresis or capillary electrophoresis. HPLC and mass spectrometry are commonly used methods for the identification of molecular weight, which should be consistent with the theoretical values. To determine the amino acid sequence of the target product, a comprehensive method is needed to verify the theoretical amino acid sequence corresponding to its gene sequence. The peptide coverage should be 100%, and the terminal amino acid sequence should be completely consistent with the theoretical sequence. Mass spectrometry methods are now the most widely used for sequencing and identifying proteins. Edman degradation is a valuable tool for characterizing the protein's N-terminus. The target protein was treated with 1% acetic acid solution, the N-terminal amino acid sequence was sequentially cut and analyzed by high-performance liquid chromatography [77]. Infrared spectroscopy is also commonly used to provide information on the chemical composition of recombinant collagen. The ion exchange chromatography with the post-column derivatization method was used to analyze the amino acid composition of the samples [78]. The results of the amino acid sequencing of the recombinant collagen should include the linking mode of the disulfide bond, and the following situations: (i) N-terminal methionine (products expressed by the *E. coli* system); (ii) a leader sequence or signal peptide; (iii) other possible N-terminal and C-terminal modifications, such as C-terminal processing, N-terminal pyroglutamate, amidation, partial degradation by exopeptidases and acetylation; and (iv) various other heterogeneities (such as disulfide bond mismatches, fragmentation, oxidation, isomerization, amidation, O-linked and N-linked oligosaccharides and aggregation, glycosylation).

### Structure characterization of recombinant collagen

**Figure 8.**
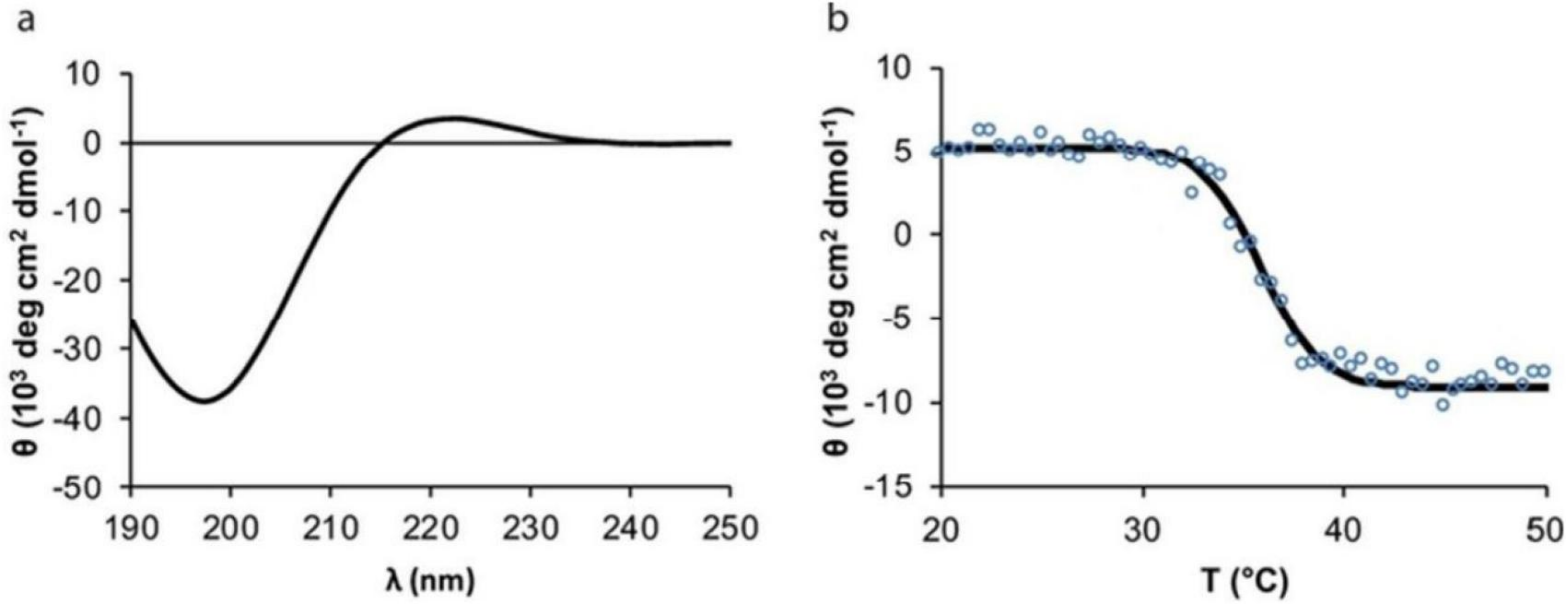
Typical circular dichroism spectrum of recombinant collagen with a triple helix structure; the left is the wavelength scanning spectrum, and the right is the temperature scanning spectrum. Reproduced from [80] with permission of John Wiley & Sons, Inc., © 2023.

Amino acid sequence analyzer or mass spectrometry could be used for the characterization of the primary structure of recombinant collagen. The triple helix structure of recombinant collagen was usually analyzed by using circular dichroism chromatography, micro-differential scanning calorimetry (DSC) and specific protease-sensitive detection methods. Que *et al.* [79, 80] used circular dichroism spectroscopy to characterize the triple-helix structure of recombinant collagen-like proteins that were used to construct recombinant collagen scaffolds for human neural stem/progenitor cells. If the collagen-like protein molecules possess a triple-helix secondary structure, the CD wavelength scanning spectrum would show a positive peak at 221 nm and a negative peak at 198 nm, which are associated with the polyproline II conformation of the collagen-like molecule. Vitale *et al.* [81] studied the chimaerism of native and recombinant collagens within a Fmoc-based self-assembled hydrogel, using CD to characterize the secondary structures of the recombinant collagen. All circular dichroism spectra of natural rat tail collagen, collagen-like peptide (GFOGER) and recombinant collagen (DCol1) exhibited triple helix structure characteristics (Figure 8). The temperature scanning spectrum with the maximum positive ellipticity at a wavelength of ∼221 nm can also reveal the thermal stability of the recombinant collagen, as shown in Figure 8.

In addition, optical tweezer single-molecule stretching, atomic force microscopy and transmission electron microscopy can be used to detect the structure of recombinant collagen. Wieczorek *et al.* [38] characterized the structure and self-assembly process of synthetic full-length recombinant human type II procollagen by mammalian expression system, using both aforementioned methods and circular dichroism.

### Controlling the purity and impurities in recombinant collagen

During the clinical application of recombinant collagen, various side effects, including allergic reactions and cytotoxicity, may result from impurity residues of the products. Therefore, the purities and impurities in recombinant collagen should be researched and clarified to assess their risk acceptability. The purity could be identified by chromatography or electrophoresis, which have been broadly applied for protein identification. The main impurities in recombinant collagen products include the presence of residual exogenous DNA, host cell protein and peptidoglycan, bacterial endotoxin and antibiotics from fermentation culture system and additives in subsequent production processing, such as enzymes, chemical/biochemical treatment reagents (cyanogen bromide, guanidine, oxidants and reducing agents), inorganic salts (heavy metals, arsenic and nonferrous metal ions), solvents, carriers/ligands (monoclonal antibodies) and other filterable substances.

Ultraviolet (UV) spectrum detection is a quick method to detect the existence of miscellaneous proteins in the recombinant collagen product. Collagen containing no tryptophan and a very small content of tyrosine exhibits strong absorption below 230 nm in the UV spectrum, in contrast, general proteins generate a strong absorption peak at 280 nm due to the unique benzene ring structure of tryptophan and tyrosine. As a result, the differentiation between 280 and 200–230 nm in the UV spectrum could be utilized to monitor miscellaneous protein contents in collagen products [82]. Other additive residues could be tested quantitatively and qualitatively by using high-performance liquid chromatography (HPLC). For instance, He *et al.* [83] used HPLC to evaluate the residues of the cross-linking agent EDC/NHS and solvent ethanol in a recombinant collagen-based hemostatic sponge with good biocompatibility and hemostatic effect.

## The prospect of genetic engineering technology to prepare recombinant collagen seed cells

### Physical transfection

Physical transfection refers to breaking the cell membrane temporarily through mechanical action or external physical field induction to deliver the required molecules into the host cell [84]. Currently, the commercial transfection methods are insufficient to meet the requirement of cell viability and transfection quantity of host cells, especially animal cells (such as CHO cells) [85]. Therefore, large-scale, low-cost, sustainable and highly versatile transfection platforms for genetic engineering and practical applications are urgently needed. Physical transfection combined with micro-nano technology has made breakthroughs in improving transfection accuracy, quality and flux [86].

With the rapid development of a series of micro-nano manufacturing technologies such as lithography, reactive ion lithography and Plasma Enhanced Chemical Vapor Deposition (PECVD), the preparation of large-area ordered micro-nano structures was gradually realized with great scalability and high-throughput potential. McKnight *et al.* [87] successfully achieved GFP plasmid transfection into CHO cells by PECVD-produced chemical modification of microneedle with a length of 6–10 μm and a tip diameter of 20–50 nm. Kim *et al.* [88] prepared high-aspect-ratio silicon nanowire arrays, allowing the growth of cells on the surface by sedimentation to achieve plasmid transfection.

Besides, due to the advantages of continuous, large-scale and low cell damage transfection of nanoneedle arrays, a large number of cells can be cultured on nanoarrays, achieving *in situ* observation through the analysis methods such as fluorescence microscopy, at the same time. For example, Shalek *et al.* [89] verified the transferability of various small molecules including DNA, RNA, protein and polypeptides to host cells such as Hela and CHO by attaching cells to the chemically modified silicon nanowire array with the tiling technique. The GFP plasmid was successfully transfected to cells by stably culturing cells on hollow aluminum nanowire arrays, combined with a microfluidic system at the bottom to achieve continuous cell transfection [90].

Moreover, to achieve effective high-throughput cell transfection, it is necessary to control the touch and separation process of the cell-microneedle array, combined with a microfluidic platform to achieve continuous cell transfection. The nanoneedle array is driven by the piezoelectric oscillation performed by micron-scale vertical vibration for cell transfection, which effectively transfected HEK293 cells with NIH3T3 and Cre recombinase [91]. Besides, large-scale, densely packed and ordered microsphere arrays were prepared by the self-assembly method and reactive ion lithography, finally achieving high-throughput and efficient NK cell transfection through microneedle-vibration and verifying the feasibility of its high gene knockout efficiency.

Compared to the traditional transfection methods such as liposome transfection and electro-transfection, the microneedle arrays are universal for a variety of gene carriers [92] and exhibit superiority in transfection efficiency and cell survival rate, especially for immune cells and animal cells [91]. With the development of micro–nano manufacturing and microfluidic technology, the introduction of fine and orderly micro–nano structures can achieve higher precision cell manipulation in micro-continuous flow. By precisely controlling the microneedle array-cell contact-detachment process, high-throughput and high-efficiency cell transfection can be achieved, which has great application potential in the field of large-scale production of recombinant collagen, especially in the construction of animal cell expression systems producing highly active recombinant human collagen with natural like structure.

Micro-injection system is another method for the efficient transfer of the target nucleotide with controlled amounts into the nucleus of a specific host cell. the transduction efficiency of single-cell microinjection is much higher compared to other methods, which could theoretically be close to 100% in the viable and successfully transfected cells [92, 94]. However, this method is still not very suitable for a large number of cells transfection, because of the difficult operation process.

### Magnetic cell sorting

In recombinant collagen engineering, commonly used physical sorting methods such as gradient centrifugation or cell filtration cannot evaluate the gene editing results of host cells due to the low physical variability of different transfection-degree cells. Biological methods, targeted to the proteins of transfection cells, are more reliable and that is why magnetic cell sorting is an optimal purification method for collagen engineering cells. Magnetic cell sorting technique has been broadly used in immune cells, tumor cells and even bacteria, due to its high specificity and biocompatibility, showing great application potential in cell recognition and sorting, positioning capture, precise control and enriched purification. For example, Wang *et al.* used magnetic cell sorting technology to efficiently separate tumor-infiltrating lymphocytes (TILs), which were marked with magnetic nanoparticles and then sorted by magnetic force through a microfluidic device. Compared with traditional cell sorting methods, the immunomagnetic cell sorting method exhibited a higher sorting efficiency, accuracy and specificity. Besides, the high diversity of TIL cells separated by this method accelerated TIL expansion and enhanced their therapeutic potential [95]. In another study, the Prism Chip (a high-resolution immunomagnetic profiling and separation chip) was applied to sort circulating tumor cells (CTCs), which can obtain exceptional cell purity and realize the identification and sorting of CTCs in whole blood samples with a large amount of red blood cell interference [96]. The microfluidic chip technology was also used for the identification, sorting and manipulation of the localization of *E. coli*, which were introduced to the chamber for culture medium screening [97].

**Figure 9.**
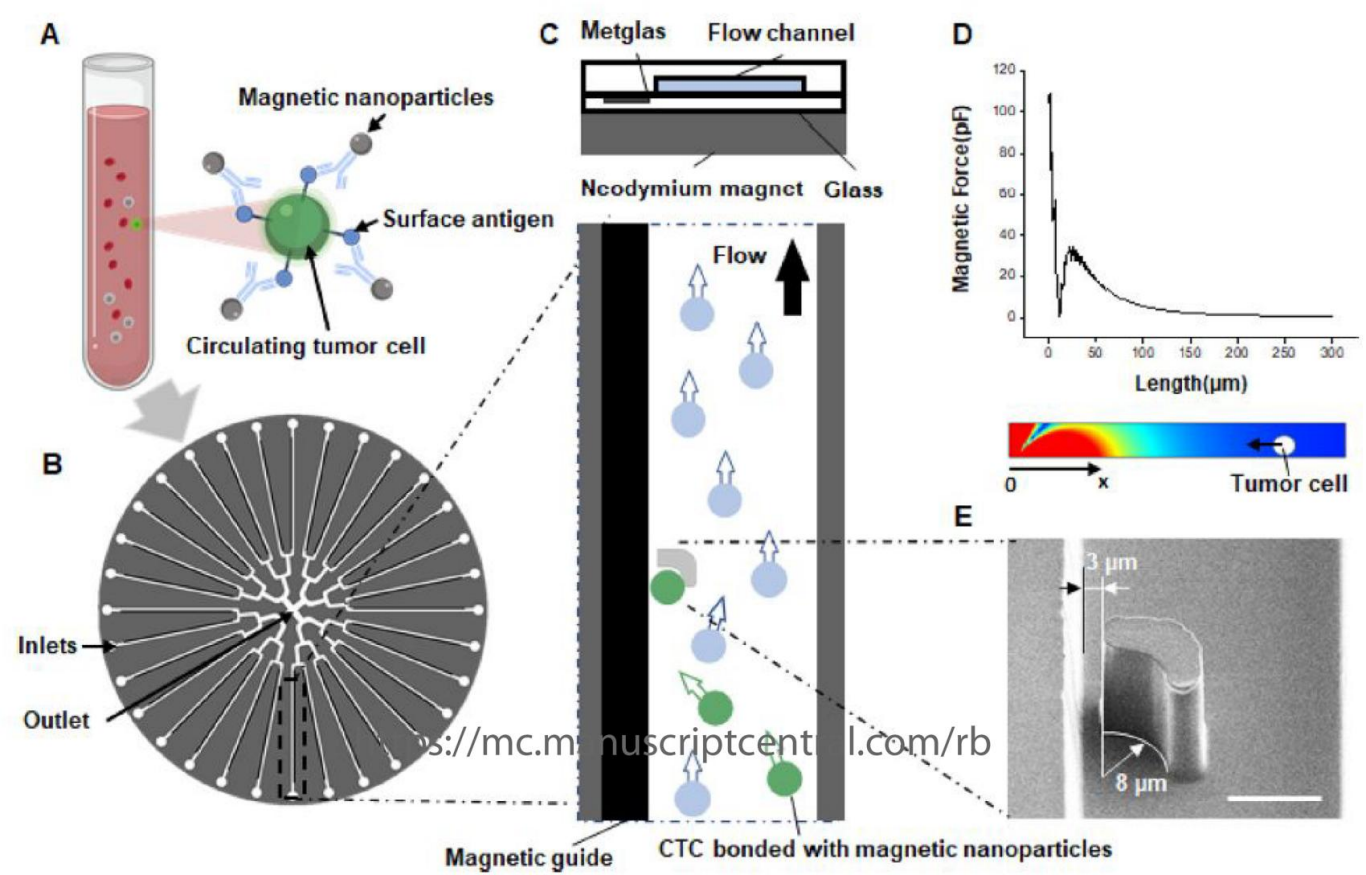
Schematic of the SCP chip for the CTC adhesion and metastasis. (**A**) Target cells are labeled with antibody-conjugated magnetic particles in a buffer or processed blood samples. (**B**) The overall architecture of the SCP chip. (**C**) Cell capture approach used in the SCP chip. (**D**) Calculated magnetic force distribution along *X*-direction when magnetically labeled cells flow through the constriction channel. The top graph shows the values of magnetic force as a function of distance and the bottom image displays this information as a heatmap. (**E**) Representative SEM image of the capture area. The scale bar is 10 μm. Adapted from [98] with permission of American Chemical Society, © 2021.

As shown in Figure 9, Ma *et al.* [98] obtained the target subpopulation cells by the specific binding of magnetic beads-antibodies to membrane antigen proteins and consequently achieved directional motion control, efficient sorting and collection of specifically labeled cells through the optimal design of the three-dimensional magnetic field. During the process of cell magnetic labeling, the amount of magnetic nanoparticles modified to the cell surface is closely related to the specific membrane protein phenotype of the cell, indicating that the resulting difference in cell magnetism can be used to analyze the membrane protein phenotype. Poudineh *et al.* [99] used magnetic nanoparticles to label cancer cells such as MCF-7, SKBR3 and PC-3 and then used gradient capture magnetic fields to analyze protein expression and distribution to achieve cell phenotype analysis. Compared with drug screening, fluorescent labeling, limiting dilution, etc., the use of microfluidic field coupling magnetic field for cell magnetic manipulation can realize high-sensitivity and high-throughput cell sorting, which is expected to be no less than one million levels per minute. Besides, the magnetic strength reflecting the cell phenotype can be also used for the evaluation of the transfection effect, improving the construction efficiency of the recombinant collagen expression system due to the advantages of short pretreatment time, high binding reliability, strong stability and large-scale application.

In summary, compared with flow cytometry sorting technology with only tens of thousands of sensitive thresholds, the magnetic sorting method exhibits higher sensitivity, throughput and specificity and can identify manipulated cells at the single-cell level. The sorting stability resisted various potential interfering factors, such as the large number of cells without specific protein phenotypes in the system, the surface charge of the channel, solution pH and ion concentration [100]. Therefore, magnetic cell sorting could be a good candidate, for use in the construction of the complex expression system for recombinant collagen production.

## Conclusions and future perspectives

Recombinant collagen has broad application potential in the fields of healthcare products, cosmetics and especially biomedicine due to its excellent biocompatibility, controllable quality and low virus risk. However, many problems limit the large-scale application of recombinant collagen, mainly the insufficient post-translational modification of recombinant collagen and low yield. Post-translational modifications, especially hydroxylation, are still a major hindrance in prokaryotic expression systems. Even in eukaryotic expression systems, recombinant collagen cannot possess sufficient hydroxylation as the natural collagen molecules. Therefore, highly effective co-expression of collagen and hydroxylase is necessary for the production of recombinant collagen similar to natural human collagens. There are also some problems in the market supervision and access of recombinant collagen products. Supervision agencies have not reached a clear consensus on the large-scale production and clinical application of recombinant collagen products, which seriously impedes the commercialization of recombinant collagen products, especially in the biomedicine field.

Up to date, the production cost of recombinant collagen is inferior to that of tissue-derived collagens. The development and application of a wide range of new science and technology will drastically reduce the research and development time and cost of recombinant collagen and produce recombinant collagen with lower price, higher output and better bionics. For example, the preparation process of functional plasmids has become easier and cheaper due to the rapid development of gene editing technology (such as CRISPR). The development of high-efficiency plasmid transfection techniques has provided powerful tools for the production of foreign proteins in various host cells (from bacteria to mammalian cells). Advances in bioinformatics, such as more advanced codon optimization algorithms, would give great help in increasing the yield of recombinant collagen. Consequently, all these progresses will realize the production of new recombinant collagen raw materials with high diversity, clinical safety and a variety of biological functions, further expanding their application in the biomaterials or biomedical industries.
